# Maternal butyrate supplementation induces insulin resistance associated with enhanced intramuscular fat deposition in the offspring

**DOI:** 10.18632/oncotarget.14375

**Published:** 2016-12-30

**Authors:** Yanping Huang, Shixing Gao, Jinglong Chen, Elke Albrecht, Ruqian Zhao, Xiaojing Yang

**Affiliations:** ^1^ Key Laboratory of Animal Physiology & Biochemistry, Nanjing Agricultural University, Nanjing 210095, P. R. China; ^2^ Leibniz Institute for Farm Animal Biology, Institute for Muscle Biology and Growth, Dummerstorf, Germany

**Keywords:** maternal butyrate, insulin resistance, epigenetic regulation, offspring skeletal muscle

## Abstract

Maternal nutrition is important for the risk of the offspring to develop insulin resistance and adiposity later in life. The study was undertaken to determine effects of maternal butyrate supplementation on lipid metabolism and insulin sensitivity in the offspring skeletal muscle. The offspring of rats, fed a control diet or a butyrate diet (1% sodium butyrate) throughout gestation and lactation, was studied at weaning and at 60 days of age. The offspring of dams fed a butyrate diet had higher HOMA-insulin resistance and impaired glucose tolerance. This was associated with elevated mRNA and protein expressions of lipogenic genes and decreased amounts of lipolytic enzyme. Simultaneously, enhanced acetylation of histone H3 lysine 9 and histone H3 lysine 27 modification on the lipogenic genes in skeletal muscle of adult offspring was observed. Higher concentration of serum insulin and intramuscular triglyceride in skeletal muscle of offspring from the butyrate group at weaning were accompanied by increasing levels of lipogenic genes and enrichment of acetylation of histone H3 lysine 27. Maternal butyrate supplementation leads to insulin resistance and ectopic lipid accumulation in skeletal muscle of offspring, indicating the importance of short chain fatty acids in the maternal diet on lipid metabolism.

## INTRODUCTION

Maternal nutrition during gestation and lactation has profound effects on fetal growth and development with lifelong consequences [1, 2]. Animal models confirm that maternal diet influences the development of insulin resistance and adiposity in the offspring [3, 4]. Insulin resistance is a well-recognized factor in the development and progression of a series of disorders including type II diabetes and hypertension, dyslipidemia, and cardiovascular disease [5]. It has also been shown that nutritional factors during offspring early life possibly influence permanently the risk of an individual to develop insulin resistance in adulthood [6, 7].

Maternal dietary fatty acids not only are important as an energy source but also play important role in regulation of gene expression in cells and in intercellular communication in offspring [1, 8]. Currently, most studies focus on the effects of long-chain fatty acids rather than on short chain fatty acids (SCFA) [8]. Butyrate is a SCFA which is produced in the gastrointestinal tract [9], and acts as an energy source to meet energy requirements in ruminants and monogastric animals [10, 11]. Besides, butyrate acts as a histone deacetylase (HDAC) inhibitor which can inhibit deacetylation of histone and various transcription factors, involved in several biological pathways associated with the pathogenesis of diabetes [12, 13]. *In vivo*, several studies have shown that butyrate can alleviate high fat diet-induced obesity and improve insulin sensitivity in skeletal muscle of mice under a high fat diet [14, 15]. However, a previous study suggested maternal high fiber diet, which could produce butyrate by microbial fermentation in the gastrointestinal tract, increased the expressions of CCAAT/enhancer-binding protein (C/EBP) and peroxisome proliferator-activated receptor γ (PPARγ) related to adipogenesis [16]. Additionally, Radunz et al. [17] found that progeny from dams fed high fiber diet had increased intramuscular fat deposition and diminished insulin response. *In vitro*, butyrate was found to stimulate adipogenesis and triglyceride storage [18, 19]. Whether maternal butyrate supplementation could have effects on lipid metabolism and insulin sensitivity in the offspring skeletal muscle has never been shown.

The ectopic accumulation of lipids in skeletal muscle has been viewed as a major factor in the etiology of insulin resistance and type 2 diabetes by reducing insulin stimulated glucose uptake [20–23]. During the past two decades, increasing emphasis was placed on understanding the connection between intramuscular triacylglycerol content and insulin action. Interest in this topic grew from numerous reports showing a strong negative association between intramuscular triacylglycerol content and insulin sensitivity [24, 25]. This relationship was also evident in obese and diabetic humans as well as in several rodent and cell culture models of metabolic disease and/or chronic lipid exposure [26–28]. However, the mechanism, how maternal butyrate supplementation influences the relationship between lipid metabolism and insulin sensitivity, is largely unclear.

In the present study, we used the maternal sodium butyrate supplement during pregnancy and lactation in rat as a model to investigate the offspring skeletal muscle lipid deposition and possible consequences for the insulin sensitivity. The results will further contribute to clarify the effect of maternal short chain fatty acids on offspring metabolism.

## RESULTS

### Maternal butyrate increased offspring body and muscle weight

The body weight and gastrocnemius muscle weight (GW) (P < 0.01) of adult offspring were significantly enhanced in the butyrate group compared with control (Table 1), though both of them showed no obvious differences at weaning age.

**Table 1:** Growth performance of weaning and adult offspring of rats fed either a control or butyrate supplemented diet throughout gestation and lactation

Variables	Control (n = 8)	Butyrate (n = 8)	P-value
**Weaning rats**			
Body weight (BW) (g)	69.23±2.04	73.17±1.02	0.10
Gastrocnemius muscle weight (GW) (g)	0.46±0.03	0.48±0.02	0.43
GW : BW (g/kg)	6.60±0.24	6.60±0.26	0.99
**Adult rats**			
Body weight (BW) (g)	247.89±4.51	288.87±7.78	<0.01
Gastrocnemius muscle weight (GW) (g)	2.92±0.06	3.37±0.12	<0.01
GW : BW (g/kg)	11.18±0.64	11.66±0.27	0.52

The values are presented as mean ± SEM.

### Impaired insulin sensitivity in offspring of butyrate supplemented rats

**Figure 1:**
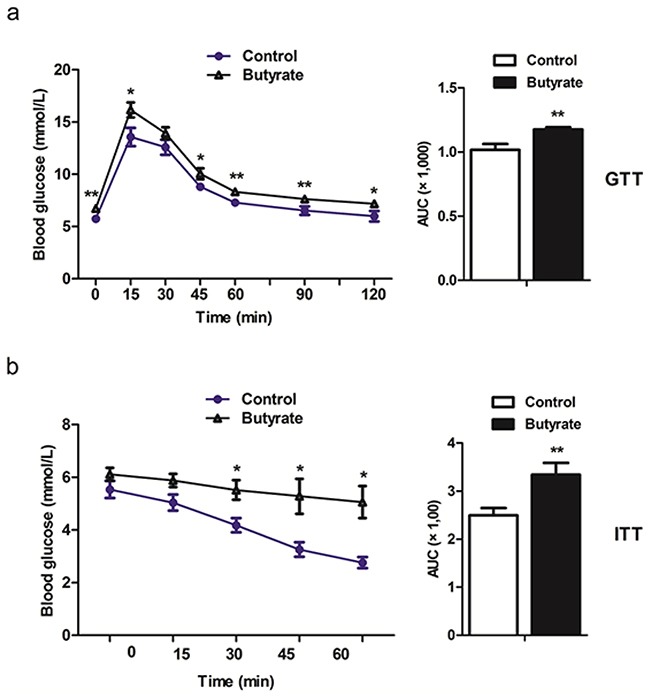
Maternal butyrate supplementation throughout gestation and lactation induced the whole body insulin resistance as indicated in glucose tolerance test a. (n = 10 in controls; n = 12 in butyrate group) and insulin tolerance test b. (n = 5 per group) in adult offspring *P < 0.05, **P < 0.01, compared with control.

Serum concentrations of insulin and NEFA were increased (P < 0.05) at weaning in offspring of butyrate supplemented rats (Table 2). This difference was not observed in adult offspring. However, concentration of fasting glucose was higher (P < 0.05) in the butyrate group (Table 2). When adult offspring were challenged with a GTT and an ITT, butyrate group rats showed permanently increased serum glucose levels (Figure 1a, 1b), indicating decreased responsiveness as a sign for development of insulin resistance. Furthermore, HOMA-IR (HOMA-insulin resistance) was significantly increased in both weaning offspring (P < 0.01) and adult offspring (P < 0.05) belonging to the butyrate group (Table 2).

**Table 2:** Serum glucose, total triglyceride content and intramuscular triglyceride content in skeletal muscle of weaning and adult offspring

	Weaning rats			Adult rats		
	Control (n = 8)	Butyrate (n = 8)	P-value	Control (n = 8)	Butyrate (n = 8)	P-value
Serum Glucose (mmol/L)	7.21±0.26	6.84±0.16	0.80	10.36±0.77	13.03±0.76	<0.05
Serum Insulin (μU/ml)	10.87±2.30	16.41±1.96	<0.05	13.32±1.46	16.61±2.35	0.20
Serum Cholesterol (mmol/L)	2.98±0.18	2.93±0.09	0.22	1.51±0.06	1.77±0.27	0.44
Serum Triglyceride (mmol/L)	2.22±0.48	1.82±0.21	0.79	2.08±0.44	1.84±0.17	0.89
Serum NEFA (μmol/L)	441.6±36.3	634.2±55.0	<0.05	590.3±28.5	569.3±56.4	0.73
IntramuscularTriglyceride (mg/g)	1.99±0.18	3.50±0.49	<0.05	1.46±0.67	4.38±1.08	<0.05
HOMA-IR	2.29±0.23	4.87±0.46	<0.01	3.31±0.42	4.89±0.66	<0.05

The values are presented as mean ± SEM.

### Maternal butyrate inhibited protein expressions related to the insulin signaling and glucose uptake n skeletal muscle from adult offspring

**Figure 2:**
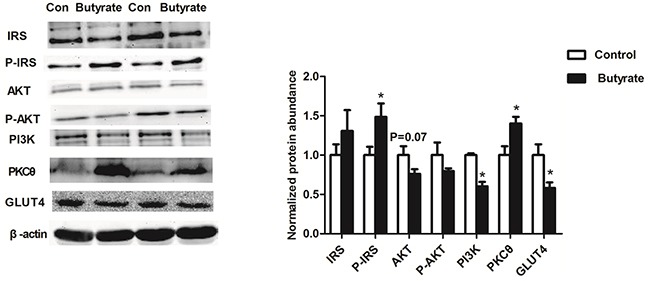
Effect of maternal butyrate supplementation throughout gestation and lactation on protein expression of insulin signaling molecules and GLUT4 protein expression in skeletal muscle of adult offspring (n = 6 per group) β-actin was used for normalization. Values are mean ± SEM. *P < 0.05, compared with control.

Because of the whole body insulin resistance observed in the adult offspring, we examined components of the insulin-signaling pathway in these animals. Phosphorylation of IRS at ser1101 was increased (P < 0.05) and the abundances of total PI3K/P85 (P < 0.05) and total AKT protein (P = 0.07) were reduced in skeletal muscle of the butyrate group (Figure 2). With the higher concentration of fasting glucose in adult rats, GLUT 4 related to glucose uptake in skeletal muscle was decreased (P < 0.05) in the butyrate group (Figure 2).

### Maternal butyrate increased intramuscular triglyceride content of offspring

Serum total cholesterol and total triglyceride were not affected in the offspring at weaning by butyrate supplementation of the dams (P > 0.05). However, significantly higher (P < 0.05) content of intramuscular triglyceride was observed in the butyrate group at weaning (Table 2).

Later in life, total cholesterol and triglyceride in serum were also not different between the butyrate group and the control group, while the content of intramuscular triglyceride further increased significantly (P < 0.05) in the butyrate group (Table 2). Oil-red O staining revealed the abundance of intramuscular adipocytes in muscle cross sections of butyrate group rats but not in that of controls (Supplementary Figure 1).

### mRNA and protein abundance of lipid metabolism related genes in gastrocnemius muscle

**Figure 3:**
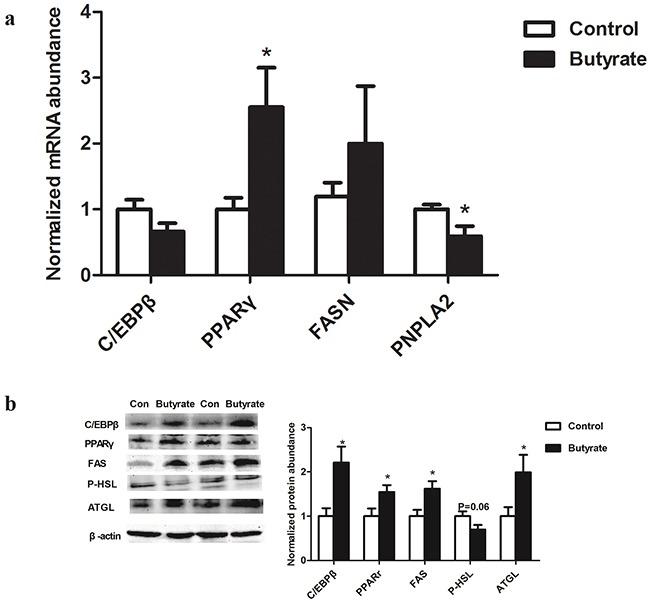
Effect of maternal butyrate supplementation throughout gestation and lactation on mRNA **a**. (n = 8 per group) and protein expression **b**. (n = 6 per group) of genes related to lipid metabolism in skeletal muscle of weaning offspring β-actin was used for normalization. Values are mean ± SEM. *P < 0.05, compared with control.

Among these lipid related genes, PPARγ mRNA expression was higher (P < 0.05) in skeletal muscle of weaning rats in the butyrate group, which was consistent with a significant up-regulation of PPARγ protein expression (P < 0.05). Although C/EBPβ and FAS mRNA expressions in weaning rats were not altered, their protein levels were higher (P < 0.05) in the butyrate group. On the other hand, PNPLA2 (ATGL) mRNA expression related to lipolysis was significantly decreased (P < 0.05) (Figure 3a) while its protein level was still higher (P < 0.05) in weaning rats of the butyrate group (Figure 3b).

**Figure 4:**
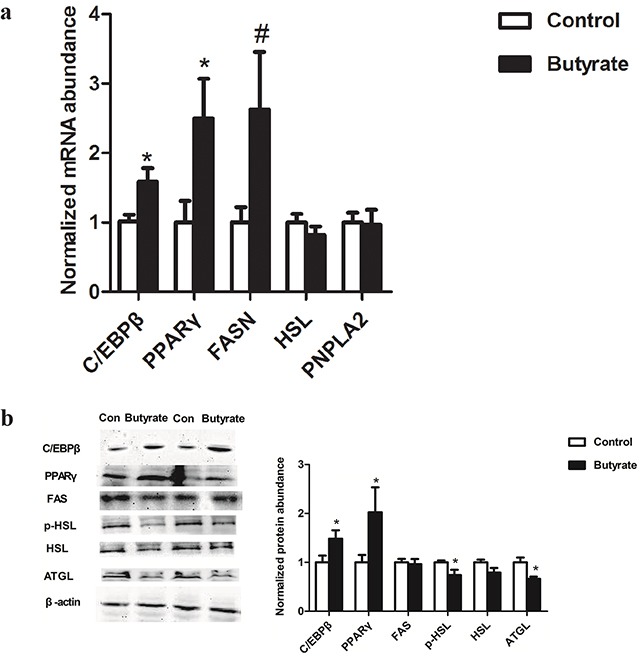
Effect of maternal butyrate supplementation throughout gestation and lactation on mRNA **a**. (n = 8 per group) and protein expression **b**. (n = 6 per group) of genes related to lipid metabolism in skeletal muscle of adult offspring β-actin was used for normalization. Values are mean ± SEM. *P < 0.05, ^#^P < 0.1, compared with control.

During the offspring adult life, lipid related transcription factors including PPARγ and C/EBPβ mRNA (Figure 4a) and protein abundances (Figure 4b) were enhanced (P < 0.05) in gastrocnemius muscle of female rats in the butyrate group. Among the genes involved in lipolysis, PNPLA2 and HSL mRNA expression did not change (Figure 4a) whereas the ATGL protein level (P < 0.05) and the phosphorylation level of HSL (P < 0.05) decreased (Figure 4b). FAS mRNA (Figure 4a) and protein expression (Figure 4b) in adult rats showed no obvious changes between two groups.

### Histone modifications on the promoter of lipid metabolism related genes

**Figure 5:**
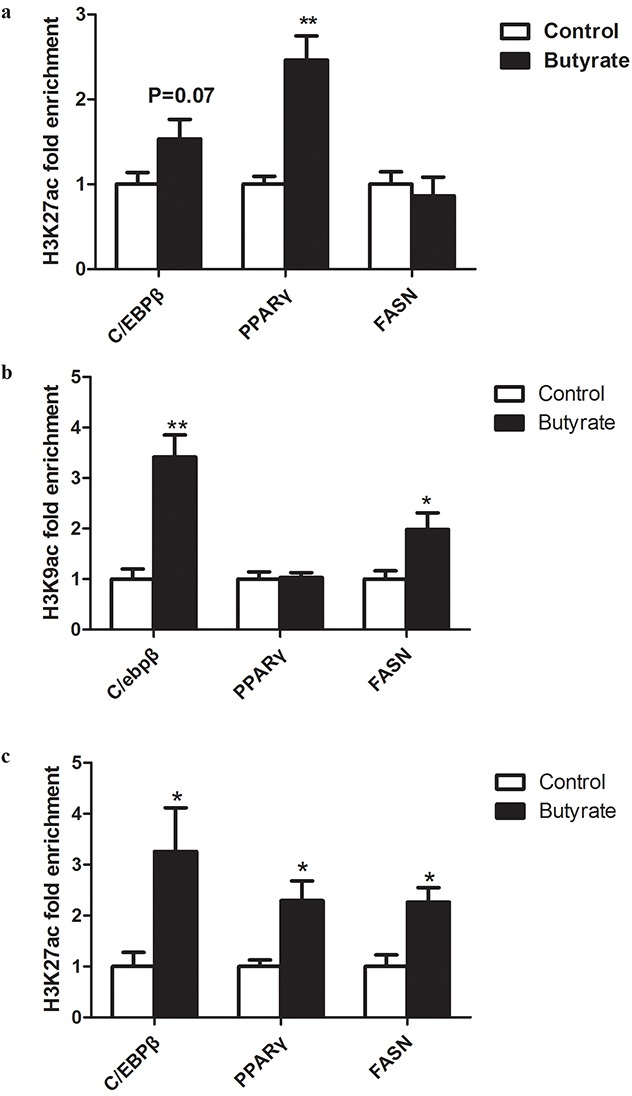
Effect of maternal butyrate supplementation throughout gestation and lactation on histone acetylation on the promoters of lipid- related genes in skeletal muscle **a**. acH3K27 enrichment in skeletal muscle from offspring at weaning. **b**. acH3K9 enrichment in skeletal muscle from adult offspring. **c**. acH3K27 enrichment in skeletal muscle from adult offspring. Values are mean ± SEM (n = 5 or 6 per group). *P < 0.05, **P < 0.01, compared with control.

In the gastrocnemius muscle of weaning offspring, higher enrichment (P < 0.05) of acH3K27 was found for PPARγ and a trend for higher enrichment (P = 0.07) for C/EBPβ in the butyrate group. Histone modification on the promoter of the FAS gene was not influenced by maternal butyrate supplementation (Figure 5a).

Furthermore, in the gastrocnemius muscle of adult offspring, maternal butyrate supplementation during gestation and lactation significantly enhanced the enrichment of acH3K9 and acH3K27 on the promoters of C/EBPβ (P < 0.05) and FAS (P < 0.05) (Figure 5b, c). Furthermore, we observed enrichment of acH3K27 on the promoter of PPARγ (Figure 5c).

## DISCUSSION

The current study highlights the importance of maternal nutrition for the offspring phenotype. It is the first to indicate that maternal butyrate supplementation during gestation and lactation induces insulin resistance in adult female offspring. The results show that insulin resistance could be induced by the accumulation of TG in skeletal muscle. Furthermore, the mechanisms involved in mediating the effects of maternal butyrate on the intramuscular fat deposition include increasing lipogenic and reducing lipolysis genes in offspring skeletal muscle partly via enhanced histone acetylation modification.

Maternal nutrition can influence the physiology and metabolism of the offspring such as insulin sensitivity [30, 31]. Prior studies have shown that offspring from dams fed high fiber diet, which could produce butyrate by microbial fermentation in the gastrointestinal tract, had diminished insulin response [17]. In the present study, the adult offspring that were born to dams on butyrate diet had higher serum glucose, HOMA-IR and impaired glucose tolerance, suggesting that they were developing insulin resistance.

Skeletal muscle is a primary site of glucose disposal and insulin action [32]. Many insulin responses are activated through the PI3-kinase/Akt pathway [33, 34]. The increased Ser1101-IRS1 phosphorylation and decreased PI3K p85α subunit and AKT showed that the insulin signaling was impaired in skeletal muscle from adult offspring in butyrate group. Increased Ser1101 phosphorylation of IRS1 has been shown to inhibit IRS1 function and could be a specific target of PKCθ [35]. The PKCθ promotes IRS-1 serine phosphorylation and impaired downstream insulin signaling [20, 35]. A higher protein expression of PKCθ was observed in skeletal muscle of adult offspring of the butyrate group in our research. Glucose uptake could be prevented by inhibiting the PI3K/Akt pathway [34]. Previous studies showed that maternal obesity during the periconceptional period resulted in lower GLUT4 protein abundance in skeletal muscle in female lambs [36]. We found maternal butyrate supplementation inhibited the protein expression of PI3K/p85 and GLUT4 in skeletal muscle in adult offspring. Our data indicate for the first that maternal butyrate diet can induce whole-body insulin resistance and inhibits insulin signaling pathway and glucose uptake in skeletal muscle in adult offspring.

Several studies have shown that a high fat diet with butyrate supplementation could alleviate obesity and improve insulin sensitivity in skeletal muscle of mice [14, 15]. However, in the present study, we observed that maternal normal diet with butyrate supplementation has increased the levels of intramuscular triglycerides in both weaning and adult offspring skeletal muscle. In addition, our previous study demonstrated maternal butyrate supplementation led to lipid accumulation in offspring liver of weaning-age rats [37]. These differences suggest that maternal normal diets with butyrate supplementation induced the onset of intramuscular triglyceride deposition in offspring at early age, indicating the importance of maternal nutrition environment.

Furthermore, in porcine stromal-vascular cells, butyrate was found to stimulate adipocyte differentiation and stimulation of triglyceride storage [18, 19]. In addition to lipid accumulation within myocytes, adipocytes developed in close proximity to myocytes, induced by upregulation of key regulating transcription factors like PPARγ and C/EBPβ [38–42]. Thus, to illustrate intramuscular triglyceride deposition, in our present study the increased expressions of C/EBPβ and PPARγ were found. Similarly, butyrate was reported to increase lipid accumulation including increasing the levels of transcriptional factors like C/EBPs and PPARγ in preadipocytes [18, 43].

Given the fact that ATGL and HSL are responsible for more than 95% of the TAG hydrolase activity [44], reduced ATGL and HSL may contribute to accumulation of intramuscular triglycerides and insulin resistance. In the present study, at adulthood, HSL phosphorylation and ATGL were dramatically reduced in the skeletal muscle of the butyrate group. At weaning age, ATGL protein expression was up-regulated and HSL phosphorylation tended to decrease with maternal butyrate supplementation. Previous studies have also shown that up-regulation of ATGL expression and/or inhibition of HSL activity promoted diacylglycerols accumulation in skeletal muscle and impaired insulin signaling [23, 45]. Taken together, the data suggests that maternal butyrate supplementation induced accumulation of intramuscular triglycerides in offspring skeletal muscle may be caused at least in part by a reduced expression of HSL and ATGL.

As shown before, butyrate has HDAC inhibitory activity [46–48]. The activation of histone acetylation could increase the transcriptional regulation of lipogenetic and metabolic genes. Previously, it has been reported that acH3K9 and acH3K27 could be activated by butyrate and enhance gene transcription [47, 48, 49]. In the previous studies, treatment of sodium butyrate accelerated adipogenesis and increased lipid accumulation by the hyperacetylation at the promoter regions of adipogenic genes such as PPARγ and C/EBP [43, 50]. In the present study, ChIP results indicated that butyrate increased the amount of acH3K9 at the promoter of C/EBPβ and the amount of acH3K27 at the promoter of PPARγ and C/EBPβ. These results support the possibility that butyrate stimulates lipogenesis in offspring skeletal muscle, in part through acetylation at the promoter regions of adipogenic genes. In this study, up-regulation of PPARγ gene was positive association with higher enrichment of acH3K27 on the promoter, in the skeletal muscle of butyrate group rats at weaning. Therefore, the increased amount of histone acetylation in the current research appears to play a role in the transcriptional regulation of lipogenic gene expression in skeletal muscle of offspring of butyrate supplemented rats. Follow-up studies *in vitro* are needed to identify the possible pathway in offspring after the maternal butyrate supplementation.

In conclusion, we are the first to demonstrate that a maternal butyrate diet during gestation and lactation leads to insulin resistance and accumulation of ectopic lipids in skeletal muscle of female offspring. This is associated with reductions in protein levels of key insulin signaling molecules and increase in protein expressions of molecules involved in lipid accumulation in skeletal muscle. These findings contribute to a better understanding of mechanisms that mediate the effects of early nutrition on the risk of developing type 2 diabetes.

## MATERIALS AND METHODS

### Experimental design and sampling

Twenty-eight virgin female Sprague Dawley rats were purchased from Laboratory Animal Centre, university of Jiangsu and housed in individual cages under temperature- and humidity controlled conditions with a 12-h light/dark cycle. After 7 d of acclimatization, rats were mated, and conception was confirmed by the presence of spermatozoids in the vaginal wash. Pregnant rats were randomly assigned to either control or butyrate diet (1% butyrate sodium), with water ad libitum. The control diet was prepared following the recommendations of the American Institute of Nutrition for rodent growth (AIN-93G) (Table 3).

**Table 3:** Nutrition standard of experimental diets

Ingredients (%)	Control	Butyrate
Corn	47	46
Wheat middling	24	24
Soya bean meal	10	10
Fish meal	4	4
Chicken meal	5	5
Premix	4	4
Salad oil	1	1
Limestone	1	1
Butyrate	0	1
Alfalfa	4	4
Total	100	100
Protein (%)	22	22
Fat (%)	6.5	6.5
Gross energy (KJ/g)	17	17

After delivering, litters were adjusted to eight animals for each dam, maintaining the sex ratio as close as possible to 1:1. During lactation, dams continued to consume their assigned experimental diet. Eight female offspring of either group were randomly selected and killed at 21 days of age (weaning). Further 8 animals per group were fed with the same control diet and killed at 60 days of age. Animals were anesthetized with pentobarbital sodium and sacrificed by bleeding. A blood sample was collected, the gastrocnemius muscles were excised, weighed, immediately snap frozen in liquid nitrogen and stored at -80°C until analysis.

All animal procedures were approved by the Institutional Animal Care and Use Committee (IACUC) of Nanjing Agricultural University. The protocol of this study was reviewed and approved with the project number 2012CB124703. The slaughter and sampling procedures strictly followed the “Guidelines on Ethical Treatment of Experimental Animals” (2006) No. 398 set out by the Ministry of Science and Technology, China and the Regulation regarding the Management and Treatment of Experimental Animals” (2008) No. 45 set out by the Jiangsu Provincial People's Government.

### Biochemical analysis

Serum concentration of glucose, total cholesterol, total triglyceride, HDL-cholesterol (HDL - C), LDL-cholesterol (LDL - C) and non-esterified fatty acid (NEFA) were measured by biochemical automatic analyzer (Hitachi 7020, HITACHI, Tokyo, Japan) using commercial assay kits (KH674, KQ436, AM545, KP712 and KF253, respectively; Wako Pure Chemical Industries, Ltd. Wako; Japan). Total triglyceride concentration in skeletal muscle (Intramuscular Triglyceride) was measured using a tissue total triglyceride assay kit (E1013; Applygen Technologies, Inc.) following the manufacturer's instructions.

Butyrate levels in the serum of the dams were published in our previous study (32.9 ± 2.9 μM vs 63.7 ± 12.3 μM; P < 0.05). It could be detected by gas chromatographic analysis. The extraction and gas chromatographic analysis procedure was carried out as described by Zhao et al. [29] with some modifications.

### Metabolic assays

Intraperitoneal glucose tolerance test (GTT) (n= 10 in controls; n = 12 in butyrate group) and insulin tolerance test (ITT) (n = 5 per group) were performed in overnight fasted rats. Blood samples were obtained from the tail tip at 0, 15, 30, 45, 60, 90, 120 min for GTT. For ITT, blood samples were collected at 0, 15, 30, 45, 60 min. Glucose levels were measured using a glucometer (AccuCheck II; Roche, Castle Hill, NSW, Australia). The doses used during these tests were 1.5 g/kg body weight and 0.5 U/kg body weight for GTT and ITT, respectively.

### RNA isolation and real-time PCR for mRNA quantification

Total RNA was isolated from gastrocnemius muscles (n = 8 per group) with TRIzol Reagent (Invitrogen Life Technologies, USA) and reverse transcribed with the PrimeScript 1st Strand cDNA Synthesis Kit (RR048A, Takara, Tokyo, Japan) according to the manufacturer's instructions. Diluted cDNA (2 µl, 1:25) was used in each real-time PCR assay with Mx3000P (Stratagene, Agilent Technologies, USA and CA). All primers (Table 4 and 5) were synthesized by Generay Biotech (Shanghai, China). For normalization, β-actin was chosen as a reference gene and was not affected by the experimental factors.

**Table 4:** Primer sequence for quantitative RT-PCR of mRNA

Gene name	Primer sequence (5′-3′)	Accession NO.	Product length(bp)
HSL	F: CGCCTTACGGAGTCTATGC	NM_012859	134
	R: TCTGATGGCTCTGAGTTGC		
PNPLA2	F: AACCAACCCAACCCTTTG	NM_001108509	179
	R: GTGGTCATCAGGTCTTTCG		
PPARγ	F: TTGATTTCTCCAGCATTTC	XM_006237009	117
	R: TGATCGCACTTTGGTATT		
C/EBPβ	F: GGGTTTCGGGACTTGATGC	NM_001301715	92
	R: GCCCGGCTGACAGTTACACG		
CD36	F: GTGCTCAACAGCCTTATC	NM_031561	218
	R: GTATCAATTATGGCAACCT		
FASN	F:CTTAGTAGTGCGTGGTCGTAT	NM_017332	195
	R: GAGTTAGCAAAGCTGGTGTC		

**Table 5:** Primer sequence for the promoters

Gene name	Primer sequence (5′-3′)	Product length(bp)
PPARγ	F: TAGGGTGGAAGGACATG	160
	R: GTTGGGAGACAGGGAAT	
C/EBPβ	F: GGTGACCGTTGCGTCCTT	115
	R: GGTAGTGGTGTCCGGGTATCA	
FASN	F: GGGACGGGAGATGATGAA	111
	R: CACTGCCCATAAGGTTGGT	

### Protein extraction and Western blotting analysis

Total cellular protein was extracted from 100 mg of frozen gastrocnemius muscle (n = 6 per group) samples as described previously [29]. Protein concentrations were measured with a Pierce BCA Protein Assay kit (no. 23225, Thermo Scientific). Western blot analysis of target proteins was carried out according to the protocols provided by the manufacturer. The sources of primary antibodies used in Western blot were as follows: anti- PPARγ (AP0686, Bioworld Technology, Nanjing, China), anti-C/EBPβ (sc150X, Santa Cruz Biotechnology, Santa Cruz, CA, USA), anti-phosphor-ser^855^ HSL (P-HSL, BS4234, Bioworld Technology); anti-HSL (BS2742, Bioworld Technology); anti-ATGL (BS7989, Bioworld Technology); anti- GLUT4 (sc53566, Santa Cruz Biotechnology); anti- phosphor-ser^1101^ IRS (BS4239P, Bioworld Technology); anti-IRS (BS3589, Bioworld Technology); anti-PI3K p85α (BS3678, Bioworld Technology); anti-AKT (AP0059, Bioworld Technology); anti-PKCθ (BS3666, Bioworld Technology); anti-Histone 3acetyl K9 (ab10812, abcam, Cambridge, UK); anti-Histone 3acetyl K27 (ab4729, abcam); β-actin (KC5AO8, Kangcheng, Nanjing, China).

### Chromatin immunoprecipitation assay

Approximately 200 mg of frozen gastrocnemius muscle samples (n = 5 or 6 per group) were ground in liquid N_2_ and washed with PBS containing protease inhibitor cocktail (no. 11697498001; Roche; Castle Hill, NSW, Australia). Cross-linking of protein and DNA was performed by adding formaldehyde to a final concentration of 1 %, and then the reaction was stopped with glycine (2.5 mol/l) at room temperature. The reaction mixture was centrifuged and the pellets were washed with PBS and lysed in a SDS lysis buffer. Chromatin was sonicated to an average length ranging from 200 to 500 bp and precleared with protein G agarose beads treated with salmon sperm DNA (40 μl, 50 % slurry, sc-2003; Santa Cruz Biotechnology). The mixture of chromatin preparations and 2 μg of specific primary antibody were incubated overnight at 4°C. A negative control was included with normal rat IgG. Protein G agarose beads (40 μl, 50 % slurry, sc-2003; Santa Cruz Biotechnology) were added to capture the immunoprecipitated chromatin complexes. Finally, reverse cross-linking was performed to release DNA fragments from the immunoprecipitated complex at 65°C for 1 h, and quantitative real-time PCR was used to quantify the fragments of target gene promoters with specific primers (Table 5) using purified immunoprecipitated DNA as the template.

### Statistical analysis

All data are presented as the mean ± SEM. The statistical analyses were performed using the Statistical Program for Social Sciences (SPSS) software 18.0 for Windows (SPSS Inc., Chicago, IL, USA). The 2^-ΔΔCt^ method was applied to analyze real-time PCR data. The differences were tested with analysis of variance (ANOVA), and a t-test was used for the independent samples. Differences were considered significant at *P* < 0.05.

## SUPPLEMENTARY FIGURE



## Abbreviations

AKT: Protein kinase B
ATGL: Adipose triglyceride lipase
C/EBP: CCAAT/enhancer-binding protein
GLUT4: Glucose transporter 4
HDL-C: HDL-cholesterol
HSL: Hormone sensitive lipase
IRS: Insulin receptor substrate
LDL–C: LDL-cholesterol
NEFA: Non esterifiable fatty acid
PI3K: Phosphatidylinositol 3-kinase
PKCθ: Protein kinase C θ
PPARγ: Peroxisome proliferator-activated receptor γ
