# Supplementary material for: Maternal butyrate supplementation induces insulin resistance associated with enhanced intramuscular fat deposition in the offspring
Source: Oncotarget. 2016 Dec 30;8(8):13073–84. doi: 10.18632/oncotarget.14375 (PMC5355078; doi:10.18632/oncotarget.14375)
Supplement: Supplementary file 1 [file oncotarget-08-13073-s001.pdf]

Maternal butyrate supplementation induces insulin resistance associated with enhanced intramuscular fat deposition in the offspring

SUPPLEMENTARY FIGURE

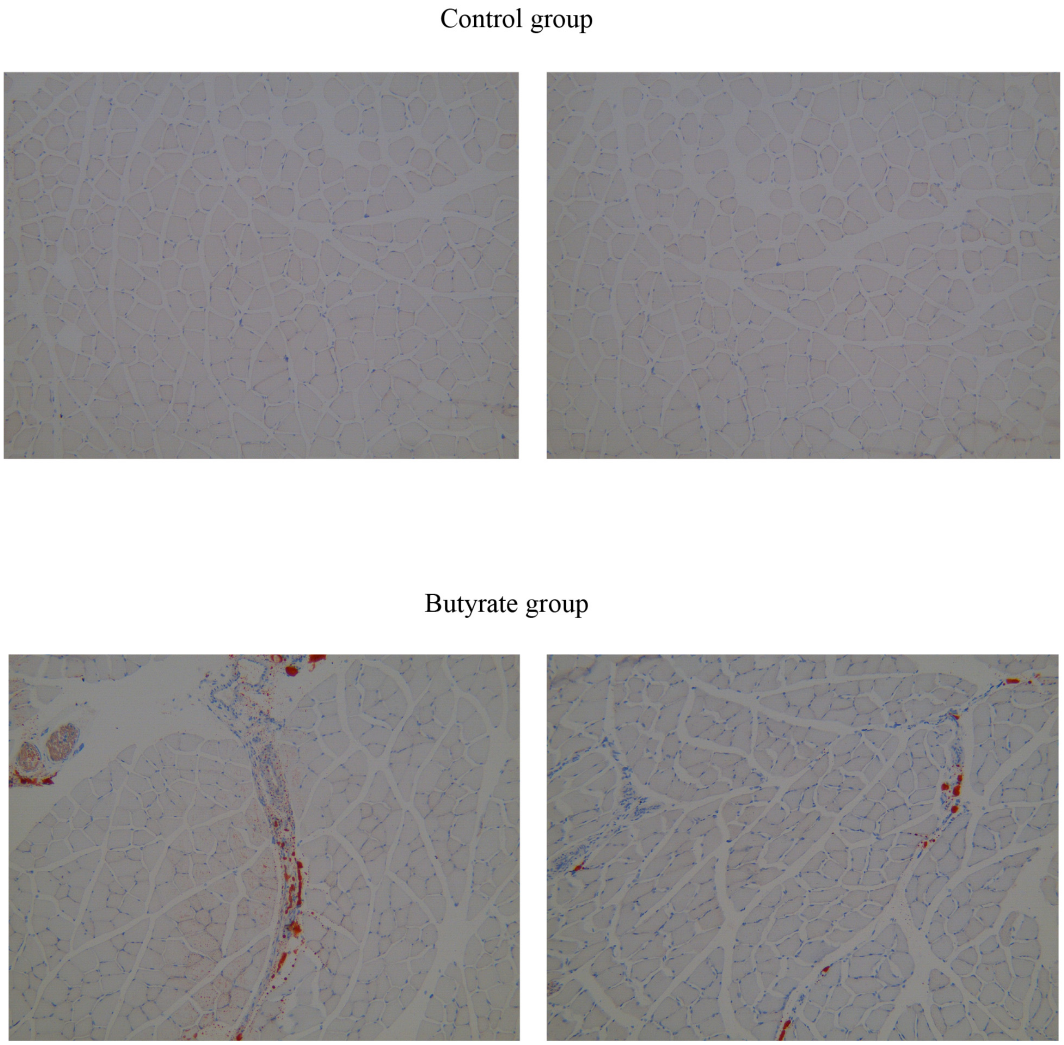

Supplementary Figure 1: Oil red o staining in skeletal muscle of adult offspring.
